# X-Ray Image-Based Real-Time COVID-19 Diagnosis Using Deep Neural Networks (CXR-DNNs)

**DOI:** 10.3390/jimaging10120328

**Published:** 2024-12-19

**Authors:** Ali Yousuf Khan, Miguel-Angel Luque-Nieto, Muhammad Imran Saleem, Enrique Nava-Baro

**Affiliations:** 1 Telecommunications Engineering School, University of Malaga, 29010 Malaga, Spain; aliyousufkhan@uma.es; 2 Institute of Oceanic Engineering Research, University of Malaga, 29010 Malaga, Spain; en@uma.es; 3 Department of Software Engineering, Sir Syed University of Engineering & Technology, Karachi 75300, Pakistan; imran@uma.es

**Keywords:** COVID, chest X-ray images, image classification, deep learning, vision transformer, lung infection

## Abstract

On 11 February 2020, the prevalent outbreak of COVID-19, a coronavirus illness, was declared a global pandemic. Since then, nearly seven million people have died and over 765 million confirmed cases of COVID-19 have been reported. The goal of this study is to develop a diagnostic tool for detecting COVID-19 infections more efficiently. Currently, the most widely used method is Reverse Transcription Polymerase Chain Reaction (RT-PCR), a clinical technique for infection identification. However, RT-PCR is expensive, has limited sensitivity, and requires specialized medical expertise. One of the major challenges in the rapid diagnosis of COVID-19 is the need for reliable imaging, particularly X-ray imaging. This work takes advantage of artificial intelligence (AI) techniques to enhance diagnostic accuracy by automating the detection of COVID-19 infections from chest X-ray (CXR) images. We obtained and analyzed CXR images from the Kaggle public database (4035 images in total), including cases of COVID-19, viral pneumonia, pulmonary opacity, and healthy controls. By integrating advanced techniques with transfer learning from pre-trained convolutional neural networks (CNNs), specifically InceptionV3, ResNet50, and Xception, we achieved an accuracy of 95%, significantly higher than the 85.5% achieved with ResNet50 alone. Additionally, our proposed method, CXR-DNNs, can accurately distinguish between three different types of chest X-ray images for the first time. This computer-assisted diagnostic tool has the potential to significantly enhance the speed and accuracy of COVID-19 diagnoses.

## 1. Introduction

In Wuhan (Hubei Province, China), officials reported an increase in cases of pneumonia with an unexplained cause on 31 December 2019. China’s Center for Disease Control and Prevention (China CDC) confirmed on 9 January 2020 that a newly discovered coronavirus (now designated as a 2019-nCoV) was the likely causal agent of these outbreaks. It has been proven by Chinese health officials that the virus may be spread from person to person. On 11 February 2020, the World Health Organization (WHO) reclassified the virus that had been circulating since 2019 from 2019-nCoV (Corona Virus Disease) to COVID-19. The coronavirus liable for COVID-19 has been officially dubbed SARS-CoV-2 (Severe Acute Respiratory Syndrome Coronaviruses) by the Coronavirus Study Group (CSG) of the International Committee on the Taxonomy of Viruses. The official classification of viruses was recently updated to include the Corona viridae family because of the novel nature of the human disease and because doing so is in conformity with phylogeny, taxonomy, and best practices. The worldwide spread and severity of SARS-CoV-2 provoked the World Health Organization to proclaim COVID-19 a pandemic [1]. Around two million people have been infected with COVID-19, making it a serious risk to health across the world. When it comes to diagnosing and keeping tabs on COVID-19 pneumonia, imaging modalities, especially CT, play a crucial role [2].

The coronavirus SARS-CoV-2, which produces COVID-19, is tested through your nose or mouth. Nucleic Acid Amplification Tests (NAATs) and antigen testing are the main viral tests. One test type may be preferred in some cases. Laboratories perform NAATs like PCR-based assays. Regardless of whether the patient is symptomatic or asymptomatic, these are the most trustworthy tests. After testing positive, viral genetic material may remain in your body for 90 days, so it is recommended that, if you are tested as positive within 90 days, you do not utilize NAATs. Antigen tests give results in 15–30 min. NAATs are less reliable when used to test symptomless people.

In this study, we address the problem of three-way classification and demonstrate that the Vision Transformer can discriminate between additional categories of lung disorders with extremely high accuracy and specificity. No other similar methods, to the best of our knowledge, have achieved such a high degree of accuracy across three distinct classes of CXR images when automatically detecting COVID-19. The major contributions of our manuscript are as follows:

- The integration of advanced techniques with transfer learning from pre-trained convolutional neural networks (CNNs);
- Our proposed CXR-DNN model’s ability to accurately distinguish between three types of chest X-ray images;
- The potential of our computer-assisted diagnostic tool to significantly improve the speed and accuracy of COVID-19 diagnosis at scale.

The structure of this work begins with examining state of the art techniques in Section 2. After that, Section 3 explains all the details of the proposed CXR-DNN technique. Results obtained with real X-rays images are presented in Section 4. Finally, the last section presents the main conclusions and remarks derived from the presented work.

## 2. Related Work

The most common data sources for subsequent detection and diagnosis of COVID-19 are CXR images, computed tomography (CT), and lung ultrasounds (LUS). All of them have been recently employed in conjunction with machine/deep learning-based (ML/DL) algorithms. In order to automatically detect COVID-19 in CXR pictures, researchers used a two steps structure involving, firstly, a CNN and, secondly, a Long Short-Term Memory (LSTM) network. In our suggested strategy, we first use convolutional neural networks (CNNs) to mine characteristics from photographs; then, we use those features to train an LSTM network to identify COVID-19 images. However, the suggested structure has its flaws. Due to its narrow emphasis on the perspective of CXR pictures, the created system had difficulty discriminating between different points of view. In addition, several unrecognized symptoms of illness were seen in COVID-19 images. The researchers came up with a strategy to spot COVID-19; they termed it COVIDetectioNet. This technique can automatically extract deep features from CXR images thanks to a pre-trained AlexNet architecture. Initially, the Relief technique was used as a feature selection strategy for selecting the most important characteristics of the image descriptors. The fast spread of the aggressive COVID-19 virus has prompted scientists from across the globe to work tirelessly on a method for early detection [3]. This was prompted by a WHO warning about the spread of the virus. It is true that stopping the spread of the illness depends on prompt detection of COVID-19 as soon as possible. In the context of this research, prompt detection involves the capacity to quickly and precisely identify COVID-19 patients from chest X-ray pictures. This method is meant to be a quick and effective screening tool, supplementing conventional diagnostic techniques such as RT-PCR, which are often slower and less available in certain environments. In emergency or resource-limited settings in particular, this approach seeks to improve diagnostic processes and provide a workable alternative for early identification by leveraging deep neural networks and properly chosen datasets. Today, RT-PCR is the golden standard for diagnosing infections. The sample is collected using devices that gain access to the nasal cavity, the oral cavity, and the posterior area of the throat, and is then analyzed in real time using molecular techniques, in particular the amplification of the viral genes that are most heavily expressed throughout the infection. This test must be performed in specially equipped labs approved by the relevant health authorities and typically takes between 2 and 6 h to obtain results. Antigen swabbing is another kind of test, although its sensitivity and specificity are much lower compared to those of molecular assays. This analysis seeks viral proteins (antigens) in respiratory specimens. Similarly to molecular testing, nose and throat swabs are used for the sample; however, the turnaround time is much faster (about 15 min). Finally, serological tests identify exposure to the virus and the presence of antibodies against it, but they can only detect an active infection in a small percentage of patients. So far, scientific studies have shown that molecular tests based on the detection of viral RNA are more accurate than serological testing [4].

In recent years there has been an increase in the use of image analysis for diagnosing infectious diseases. The most common imaging modalities for identifying COVID-19 illness are CXR and computed tomography (CT). Several methods, from automation engineering to DL [5,6,7], have thoroughly investigated the original ideas behind diagnostic imaging systems [8]. In this sense, a growing body of research has investigated how DL models can be used to automate COVID-19 diagnoses from CXR pictures. These findings are positive, but there is still a great deal of work to be conducted in terms of data collection and model development. Some research has included explanation techniques in their models to account for the possibility of bias in these kinds of challenges [9]. Differentiating between cases of COVID-19 and non-COVID-19 pneumonia (NCP) on CT was investigated using random forests with adaptive feature selection before several layers. Layer freezing and fine tuning are important to transfer learning in deep learning, especially with pre-trained models. Layer freezing preserves certain pre-trained network layers while training the rest on the target dataset. This method lets the model retain information from a larger dataset, improving its generalization to new tasks. Further fine tuning progressively unfreezes layers and trains them with a reduced learning rate to better match the target dataset. In situations with limited data, pre-trained model adaptation involves adapting a model learned on a big dataset like ImageNet for a specific purpose to save training time and improve performance. In recent years, neural networks have attracted more and more attention. A convolutional neural network (CNN) was proposed, in addition to unsupervised lung segmentation and activation maps for the area of interest localization, for the binary classification of CT scans as normal or featuring COVID-19. To better detect COVID-19, researchers investigated the use of a deep neural network (DNN) in combination with fused dynamic exemplar pyramid features [10]. CXR images are the primary focus of this study since they are easily accessible, which is not always the case with CT scans [11]. According to research [12], the sensitivity of CT scans may be higher than that of CXRs [13].

Numerous DL algorithms have been developed for the identification and diagnosis of the COVID-19 virus in medical imaging, particularly CT and CXR scans, in the aftermath of the epidemic. The CXR images have been proven to distinguish among four kinds of patients: healthy, with COVID-19, with Vir. Pneumonia, and with bacterial pneumonia. One of the first DL models to make that discrimination was COVID-Net [14], a CNN-based model. Other instances include lung disease diagnosis (classification) and organ area segmentation [15]. Recent studies on image classification with wavelet scattering networks and chest CT scans for classifying COVID-19 are discussed, as are other studies on DL for image classification [16]. Following the initial COVID-19 outbreak, a flurry of research was undertaken to identify and characterize the factors that would aid in the diagnosis of this virus. The radiological manifestations of COVID-19 on chest CT scans have been well documented in multiple studies. Moreover, research has discussed in detail the value and function of AI in identifying and treating COVID-19 patients at the screening and diagnostic stages. As a result, there have been numerous attempts to develop automated methods for identifying COVID-19 in diagnostic images like X-rays and CT scans [17].

Regarding the identification of COVID-19 in digital photographs, using a CNN is one of the most widely used and reliable techniques to identify COVID-affected lungs in X-ray pictures. In their analysis of many CNN-based results, Shazia et al. [18] highlight the 99.48% accuracy achieved by DenseNet121 in their benchmarking. The overall objective of the classification task was to differentiate between COVID and viral pneumonia. For that reason, although more than 4000 photos were available, the test set only featured 157 of them. Researchers in other papers [19,20] attempt to categorize X-ray pictures into COVID and non-COVID groups, often viral/bacterial pneumonia, or they add a third category, normal lung CXRs. However, CNNs are not the only type of neural network that can perform image recognition. Recurrent Neural Networks (RNNs) and LSTMs are two other types of neural networks that could be used for this purpose. In contrast, CNNs excel at picture analysis and are thus ideally suited for image recognition tasks due to their ability to automatically learn and extract features from input data.

Recent advances in identifying COVID-19 have been reviewed in many works. Positron emission tomography (PET), lung ultrasounds, and Magnetic Resonance Imaging (MRI) are imaging techniques also used for COVID-19 diagnosis, but the methods used depend on the patient condition and the diagnosis for the usage and treatment. We have introduced a new digital-image-reading technique by which we can obtain results more efficiently.

Another alternative is the Vision Transformer’s capabilities against those of CNNs ever since the model’s introduction. Because of its superiority over its convolutional counterparts, the Vision Transformer (ViT) is becoming popular, being employed for COVID-19 identification in a wide variety of different tasks. When comparing the ViT’s performance to that of other CNNs, Krishnan [21] found that it was able to identify between COVID-19 and non-COVID-19 chest X-ray pictures with an accuracy of 97.6%. To this model, D. Shome et al. included pneumonia as a third classification, and they also tried it with two groups. Accuracies of 92% and 98% were attained in the study, respectively. Authors provided test accuracy for each of the three groups individually, with a 95% confidence interval, the value of which is consistently superior to the regular class average of 94.7% [22]. Using the ViT, Ligi et al. [23] were able to categorize X-rays of the chest into three groups: those with chronic obstructive pulmonary disease (COPD), pneumonia, and normal lungs. All these studies involve research that primarily focuses on differentiating between those with and without COVID in addition to addressing multi-class categorization by adding viral pneumonia as a third class. To differentiate between viral and bacterial pneumonia, Almalki et al. [24] coupled their own CNN with a few hand-picked ML techniques. Even so, the best test results they could obtain with their approach reached 97.29 percent. The study presented here demonstrates the ViT’s excellent accuracy and specificity in discriminating against an additional category of pulmonary disorders, lung opacity, and extends coverage of categorization across four classes. Park et al. [25], who had previously developed a method for assessing COVID-19 severity, included a convolutional backbone for feature extraction. However, they separated X-ray pictures into three distinct groups: COVID-19, normal, and a catch-all category labeled “other illness”.

## 3. CXR-DNN Technique

The ML branch of AI is concerned with the study and creation of algorithms with the capacity for learning and adaptation. These algorithms may learn from their mistakes and become better at a certain activity without any additional instruction. ML can be used in many different fields, from natural language processing to video games. It is already making a huge difference in a wide variety of disciplines and has the potential to completely transform several established industries. The many upsides to ML include gains in effectiveness, precision, flexibility, responsiveness, automation, and economy.

**Figure 1:**
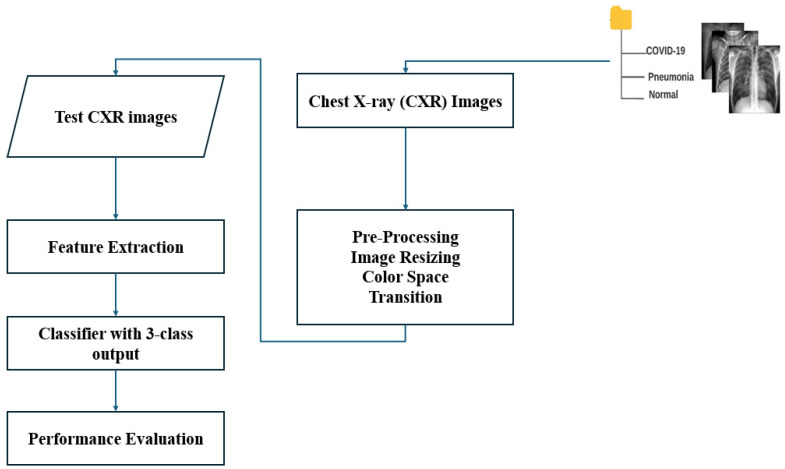
Block diagram of CXR-DNN used for screening COVID-19.

The main blocks of the algorithm ML-based CXR-DNN developed here are presented in Figure 1. This figure illustrates the CXR-DNN model’s comprehensive architecture and methodology, which entails the processing of chest X-ray images through a series of phases for the purpose of screening and classifying COVID-19, viral pneumonia, and normal cases. The diagram highlights critical components, such as data input, preprocessing, feature extraction, and final classification. The main objective is to diagnose the illness from a CXR image. Specifically, a CXR-DNN is based on an analysis of CXR images by employing a DNN.

**Figure 2:**
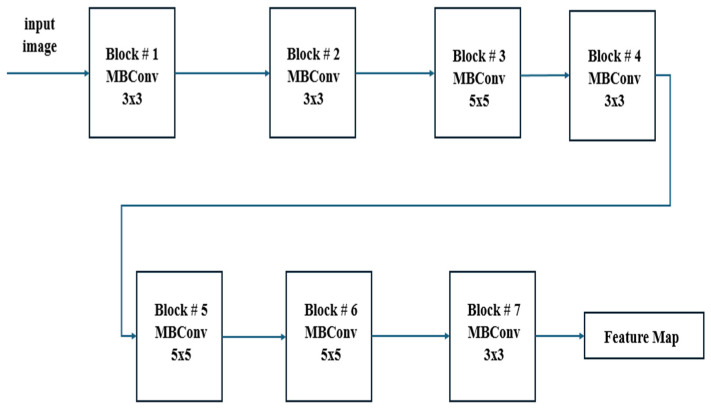
Proposed EfficientNetB7 architecture.

**Figure 3:**
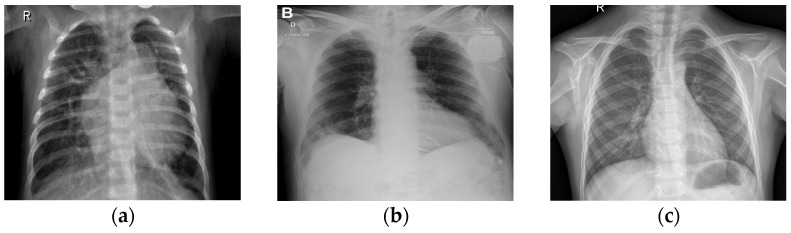
CXR images of lungs in patients: (**a**) healthy, (**b**) COVID-19, and (**c**) pneumonia.

In Figure 2, it can be observed that EfficientNetB7 uses MBConv (Mobile Inverted Bottleneck Convolution) blocks with depth-wise separable convolutions, squeeze-and-excitation, and compound scaling to efficiently balance depth, width, and resolution for high-performance image classification. This figure features detailed information regarding the EfficientNetB7 architecture employed in the CXR-DNN model. The architecture is deconstructed into its fundamental layers and modules, with a particular emphasis on the advanced convolutional and MBConv blocks that improve the precision of feature extraction and classification. EfficientNetB7’s unique compound scaling and MBConv blocks with squeeze-and-excitation make it more efficient and accurate compared to other CNN models. Since Figure 1 and Figure 2 reflect varying degrees of information within the CXR-DNN model, they are linked. Presenting the whole model’s process from input to output, Figure 1 provides a macro-level perspective. Figure 2 offers a micro-level perspective, highlighting the EfficientNetB7 architecture which is fundamental for the step of feature extraction shown in Figure 1. Taken together, they offer a complete understanding of both the general framework and the complex operations of the model. Figure 1 supports the study by visually summarizing the methodology used for COVID-19 screening, making it easier for readers to grasp the end-to-end process. Figure 2 complements this by detailing the core architecture that underpins the model’s robust performance, thus emphasizing the technical innovation and depth of the proposed solution. This combination helps to clearly convey the significance of the research and its approach to achieving reliable COVID-19 diagnostics through advanced deep learning techniques. To illustrate further the differences in CXR images belonging to classes 1 (COVID-19), 2 (normal), and 3 (pneumonia), Figure 3 shows three examples of lung X-rays (one for each class).

Firstly, the CNN divides an image into zones so that it can estimate boundary boxes and probabilities for each location [26]. The model we have developed allows us to divide our data into three categories: COVID-19, viral pneumonia, and normal. X-ray images of patients will be captured by our system and uploaded as data to an ML platform to train the model. The X-ray image will be processed by the platform, and it will be classified as best it can. Transfer learning, offering evaluation metrics, and ensuring easy accessibility and integration make it simple and affordable to acquire precise results from patients.

Next, CXR pictures were used to further train numerous pre-trained CNN models for COVID-19 detection. Subsets of classifiers with a cardinality greater than one are then aggregated using the screening of a chest X-ray [27], and their measures are calculated using the advanced proposed CNN technique. At the end, the results of three separate aggregations calculated using each of the three weighting methods (training dataset, validation dataset, and testing dataset) are evaluated in terms of confusion matrices, accuracy per class, three parameters of efficiency (precision, recall and F1-score), and, finally, graphs of the accuracy and loss per epoch. This last evaluation is the final stage of the CXR-DNN presented in Figure 1. The three images’ sets (training, validation, and testing) were images used to generate features. The photos are then labeled and placed into three categories: COVID-19, normal, and viral pneumonia. Each set’s weights are determined, i.e., training, validation, and testing.

Convolution is utilized by our proposed technique. The first component, i.e., stem block, is an attention mechanism module that processes an attention mechanism in parallel multiple times. The second component is a series of MBConv blocks that facilitate efficient feature extraction, reduced computational complexity, and enhanced representational power through depth-wise separable convolutions, squeeze-and-excitation blocks, and residual connections. The third component is compound scaling [28], which facilitates balanced model scaling by simultaneously adjusting depth, width, and resolution, leading to improved performance, optimal resource utilization, and enhanced model generalization. The next step is to determine suitable values with the help of advanced features. The accuracy is calculated according to many different performance indicators (see Figure 1).

To guarantee the dataset veracity, it was downloaded from Kaggle’s community [29]. Researchers have put together a database of CXR images, namely i.e., the COVID-19_Radiography_Dataset, for three types of patients, i.e., COVID-19, normal, and pneumonia. All pictures were saved in Portable Network Graphics (PNG) format. There are available different datasets for CXR images besides the current one we have used. We show a selection of them in Table 1.

Figure 3a shows a normal chest X-ray, which would reveal transparent lungs with no abnormalities. The consequence of suffering from COVID-19 is a fatal respiratory illness that causes severe breathing difficulties in both lungs (Figure 3b). The lungs may show patchy or consolidated opacities in a CXR with COVID-19 infection. When an infection strikes the lungs, it can lead to viral pneumonia (Figure 3c), which in turn leads to inflammation of the alveoli, the tiny air sacs in the lungs that are essential for exchanging oxygen and carbon dioxide, and which requires immediate medical advice or treatment. Coughing, fever, shortness of breath, chest pain, and exhaustion are all symptoms of pneumonia. Because of the inflammation, fluid may collect in the lungs, making it difficult to breathe. The best way to distinguish between COVID-19 and COVID-19 pneumonia is to consider them different steps of the same illness. COVID-19 is a respiratory sickness caused by SARS-CoV-2, and COVID Pneumonia is a complication of COVID-19 that causes the cited inflammation and fluid in the lungs [33]. There is reason for alarm with influenza, since it may lead to everything from a mild cold to pneumonia, acute respiratory distress syndrome, and even death.

By using tagged CXR images of COVID-19 positive, viral pneumonia, and normal cases, we trained CNN which uses advanced techniques to enhance efficiency and performance. In Table 2, the assigned class number is presented jointly with the file name used from dataset: 1 (COVID), 2 (Normal) or 3 (Pneumonia).

Upon submitting the CXR images for evaluation, we anticipate receiving the expected result, i.e., COVID-19, normal, or pneumonia. If it does not provide the predicted result based on the given class, the model will not be absolutely accurate. We have found that, for the tested photos mentioned in Table 1, our algorithm accurately returns the result for the specified class, as we will present in the next section.

## 4. Results

### 4.1. Computational and Hardware Considerations

The proposed CXR-DNN has been run on a computer with the following specifications: Intel(R) Core(TM) i5-6300U CPU (2.40 GHz), 8.00 GB RAM, and integrated graphics (Intel(R) HD Graphics 520). This system configuration stands for a modest setup and supports the system’s basic functionality for diagnostic processing. However, due to the intensive nature of deep learning algorithms, more powerful hardware such as a dedicated GPU can substantially reduce the latency and processing time, enhancing real-time diagnostic capabilities. On this system, each diagnostic run is completed in approximately in 15–60 s. This demonstrates, that while higher-end hardware is ideal for optimal performance, the CXR-DNN system can still function with more accessible lower-specification configurations.

### 4.2. Model Interpretability with Grad-CAM

We used Gradient-weighted Class Activation Mapping (Grad-CAM) to help to comprehend the CXR-DNN model and provide knowledge of its decision-making process. Convolutional neural networks (CNNs) make extensive use of this method to create visual heatmaps highlighting the areas of an input picture most impacting the prediction of the model [34]. In terms of COVID-19 viral pneumonia, or normal lung diseases, grad-CAM allowed us to pinpoint, from the chest X-ray image, predictions of the model emphasized during classification. Grad-CAM is a useful instrument for clinical application, as the pictures it shows not only boosts the confidence in the model’s predictions but also aids in improving the openness of its decision-making process. Additionally, the integration of Grad-CAM contributes to model explainability in clinical practice, helping practitioners better understand the rationale behind each prediction. This step is essential for ensuring that the model can be utilized confidently in real-world medical applications.

### 4.3. Steps of Methods on Preprocessing for Image Quality and Robustness

Improving CXR picture quality and guaranteeing model resilience in classification problems depend mostly on preprocessing methods. This work used standard preprocessing techniques, including picture scaling, normalizing, and selective augmentation. Images were rescaled to fit the EfficientNetB7 architecture’s needed input dimensions, hence fostering uniformity for efficient feature extracting. Pixel intensity levels were standardized to a [0,1] range to reduce variance and improve training stability. Additionally, rotation and flipping were judiciously employed to imitate real-world data variations, hence enhancing the generalizing powers of the model. These preprocessing techniques substantially assisted in optimizing the data for training and enhancing the dependability and diagnostic accuracy of the model in clinical situations.

### 4.4. Hyperparameter Tuning and Its Impact on Model Performance

This work aims to obtain the best performance of the suggested CXR-DNN model by means of the hyperparameter tweaking method. Based on iterative testing and field-of-expertise, we choose the learning rate, batch size, and number of epochs in deep learning. Specifically, after evaluating a variety of values (e.g., 0.001, 0.0005, 0.0001, and 0.00005), the learning rate was fixed at 0.0001 to provide sustained convergence without overshooting the minimal loss. Experiments combining computational efficiency and model generalization led us to choose a batch size of 32; lesser batch sizes (e.g., 16) resulted in noisier gradients, while higher batch sizes (e.g., 64) raised memory demand without appreciable accuracy gains. Setting the number of epochs at 100 allowed the model to acquire intricate features without overfitting, as tracked by validation accuracy, enough training time. These decisions confirmed their relevance for strong CXR image categorization by matching actual results from our pilot testing and literature benchmarks.

In this work, a CNN with advanced techniques (i.e., EfficientNetB7) with 100 epochs, a 0.0001 learning rate, and a batch size of 32 has been implemented for three classes, i.e., COVID-19, normal, and pneumonia (see Table 2). To train our algorithm for getting optimal results, we tested a total of 4035 CXR images, i.e., 1345 CXR images for each class, i.e., COVID-19, normal, and pneumonia. We have tested 70% of the dataset as trained for each class, i.e., 941 images for each of the three classes, and 15% of the dataset as validated and tested for each class, i.e., 202 images for each class. Highly accurate and low-cost automated screening of CXR images using a CNN with a transfer learning algorithm may detect COVID-19-related lung disease. Our method used random rotations and brightness changes as part of data augmentation methods to improve dataset variability and model resilience. Moreover, a fine-tuning approach was adopted wherein a lower learning rate was used when training the unfreezed layers, thereby assuring that the model could efficiently adapt to the particular properties of CXR pictures while preserving already acquired features from the pre-trained network. Overcoming previous CNN designs (e.g., ResNetXX, VGGXX) [35], this approach allowed the model to obtain a 95% average accuracy for the COVID-19 class on the training dataset. These focused approaches improve the model’s diagnostic capacity and dependability, hence facilitating real-time clinical uses. Some of the data obtained are presented in Table 3.

Results from Table 3 show that ResNet50 is the best CNN, with an 86% test accuracy, but that it is not better than ours, at 95%, mentioned above for the training dataset.

### 4.5. Evaluation Metrics and Performance Analysis

In order to make a more precise evaluation, we have used four typical efficiency parameters precision, recall, F1-score, and accuracy, for the three datasets (i.e., training, validation, and testing) of each class: COVID, normal, and pneumonia. Their definitions are given by the following usual expressions:

(1) $$P=\frac{TP}{\left(TP+FP\right)}$$

(2) $$R=\frac{TP}{\left(TP+FN\right)}$$

(3) $$F1=2\frac{P\cdot R}{\left(P+R\right)}$$

(4) $$\;\;\;A=\frac{TP+TN}{TP+TN+FP+FN}$$

(5) TN=N−(TP+FP+FN)

where *P*, *R*, *F*1, and *A* are the precision, recall, F1-score, and accuracy, respectively; *TP*, *TN*, *FP*, and *FN* are the number of true positives, true negatives, false positives, and false negatives, respectively; and *N* is the total samples used. The “macro” averaging method was employed for handling multi-class predictions in this study where precision, recall, and F1-score were calculated independently for each class. The results were then averaged to provide a comprehensive evaluation of model performance, treating each class with equal importance regardless of class frequency in the dataset, which ensures a balanced evaluation.

When we talk about genuine value in this context, we are referring to the fact that we have mentioned the classes of COVID, normal, and pneumonia; hence, if the result is exact for all three classes, then it will be truly positive (*TP*). *FN* means that we upload an image, expect its result from its mentioned class number but then find that it gives some other output. For instance, we were expecting that the generated result would be COVID-19, but if it gives some other output, it will be falsely negative (*FN*) for the COVID-19 class.

The precision denoted by *P* is the fraction (in percent) of examples where the system successfully classified the picture into that group. Another way to evaluate an ML model’s efficacy is via the F1-score. The F1-score combines a model’s precision and recall. When evaluating a model, accuracy considers all the predictions, positive and negative, with overall correct measures. In that sense, recall (*R*) is calculated as the percentage of those images that the neural network successfully classified as belonging to the specified category. Recall, on the other hand, specifies the percentage of photos successfully assigned to a class by the neural network, given the number of images that truly belong to that class. The converse is also true; in some cases, a low recall (*R*) rating is associated with a high precision (*P)* score. The total number of pictures the network has put into a certain group is the number of pictures in that group.

Table 4, Table 5 and Table 6 show the value obtained for the three parameters *P*, *R*, and *F*1 comprehensively; also, our proposed model robustness can be observed here due to its sustainability. The last column, titled “support”, shows the number of photos per category utilized to evaluate the capacity of the assigned images to the various categories. In order to compare sizes of other databases, it should be remembered that Table 1 contains an overview of the number of images in public repositories.

### 4.6. Visualization and Analysis of Confusion Matrices

**Figure 4:**
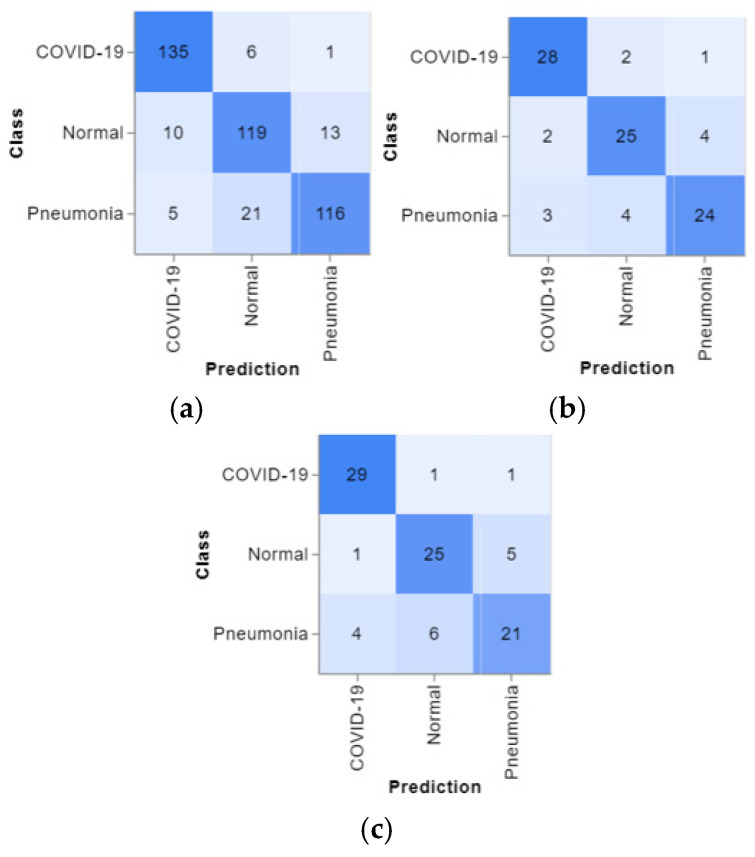
3 × 3 confusion matrices for the (**a**) Training, (**b**) Validation, and (**c**) Testing datasets representing the model’s performance in true positives, false positives, true negatives, and false negatives for each class (COVID-19, Normal, and Pneumonia).

**Figure 5:**
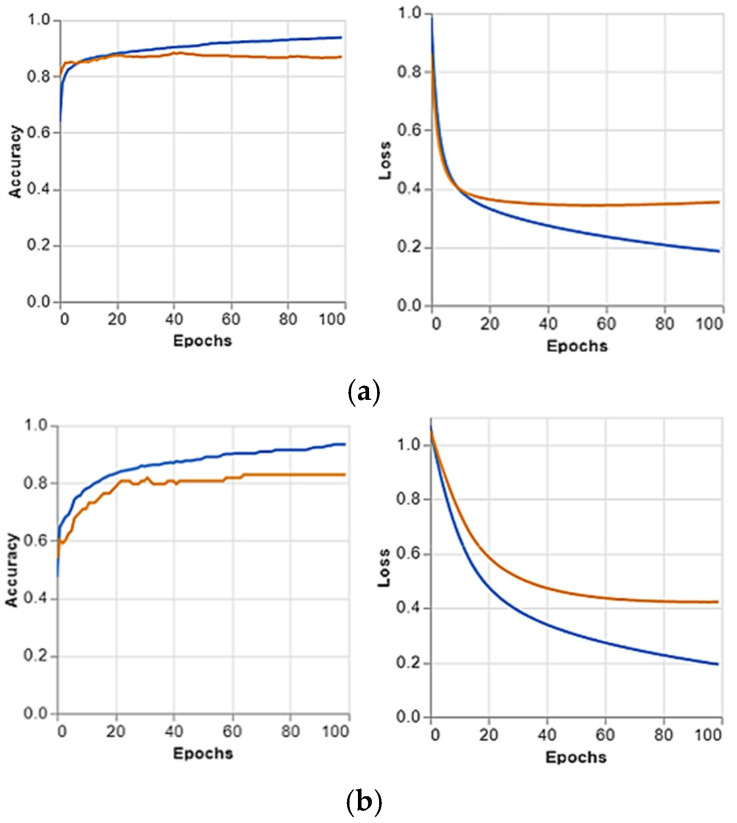

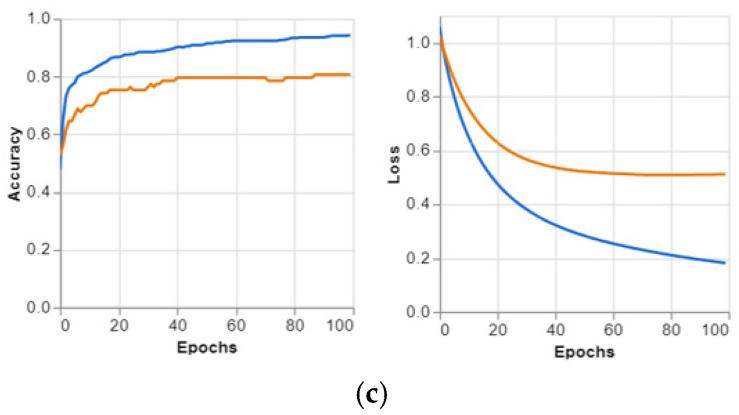
Accuracy and loss per epoch for each dataset, illustrating the model’s performance and learning progression: (**a**) Training dataset, (**b**) Validation dataset, (**c**) Testing dataset. In the accuracy graphs, the blue curve represents accuracy, and the orange curve represents loss. In the loss graphs, the blue curve represents loss, and the orange curve represents accuracy, showing their variation over the epochs.

The confusion matrices are presented in Figure 4, and the predicted positive images of CXR can be observed very clearly in them. These matrices focus on the model’s classification performance across COVID-19, pneumonia, and normal chest X-ray images. Given the similarity in radiographic features between pneumonia and COVID-19, such as ground glass complexities, there is an essential overlap between the two classes. This overlap might result in misclassifications, specifically false positives or false negatives, as the model may struggle to differentiate these conditions correctly. The accuracy per epoch and loss per epoch are shown in Figure 5 for every dataset used. In all of them, the accuracy per epoch is close to 1, whereas the loss per epoch is close to 0, which indicates a high reliability and robustness in our proposed CXR-DNN model.

### 4.7. Performance Metric Convergence Graphs

**Figure 6:**
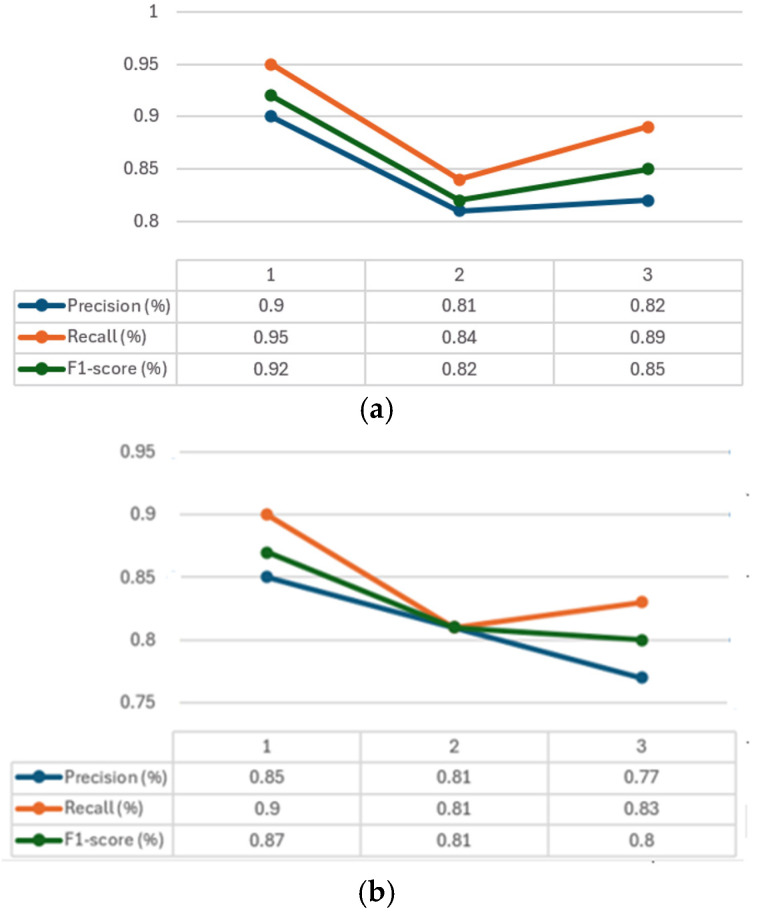

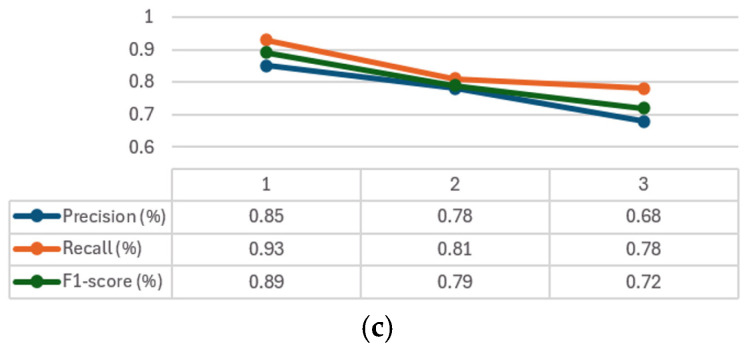
Convergence for precision, Recall and F1-score in every dataset used: (**a**) training, (**b**) validation, (**c**) testing. Classes: 1—COVID-19, 2—normal, 3—pneumonia.

The convergence graphs of precision, recall, and F1-score are presented in Figure 6 for every class considered (1–3). It can be seen how, in all the graphs (Figure 6a–c) CXR-DNNs assess a good class’s prediction accuracy. The best accuracy score (*p* = 95%) using our proposed model CXR-DNN is shown in Table 7. These results imply that the network may benefit from transfer learning to extract useful characteristics in terms of COVID-19 illness diagnosis. More than one piece of work has used this concept to speed up the creation of a trustworthy instrument to aid medical professionals in the diagnosis of COVID-19.

**Figure 7:**
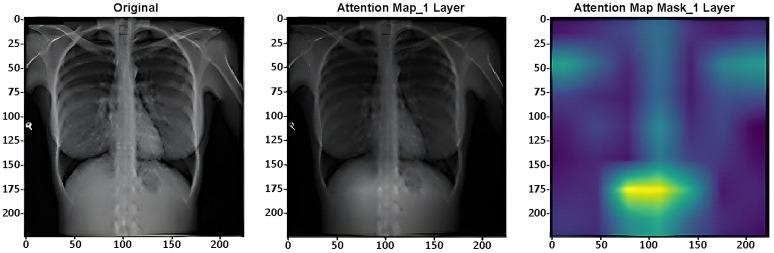
Vision sample COVID-19 CXR picture with transformer attention map (layer 1).

Attention maps are used for assessing the performance of DL systems on image categorization. As example, Figure 7 presents one layer (of 4) of the ViT’s layers [36] in the CXR-DNN proposed.

## 5. Discussion

The results reported so far have shown that the proposed architecture can outperform alternative network configurations for this unusual application. If the illness of interest is the root cause of the present pandemic, then a rapid, accurate, and reliable approach to diagnosing lung infections quickly takes on even greater relevance. While the EfficientNetB7 architecture has shown good performance in regard to COVID-19 predictions using chest X-ray images, on the other hand, there are several limitations that should be acknowledged, such as its effectiveness being highly dependent on the quality of the dataset, as any bias or inadequacies can adversely affect the model’s generalization capabilities. In addition, EfficientNetB7 may require necessary computational resources which may not be available in clinical environments. This architecture has been designed for precise differentiation between COVID-19 and other respiratory conditions and is very important for rapid and automated assessments in high-demand healthcare situations. Future work should address limitations related to dataset diversity and model generalizability. Furthermore, this approach shows the potential for adaptation to related medical imaging tasks, such as detecting other thoracic diseases, thereby broadening its utility for clinical decision-making capabilities.

This model also operates as a black box, making it challenging to represent the decision-making process. While it performs well on the data used, its scalability to different situations in chest X-ray imaging techniques may require essential further tuning and refining. Finally, the current architecture is tailored for COVID-19 diagnoses and may not be as effective for other thoracic pathologies without verification and validation. The opaque nature of DL and neural networks generally makes it challenging to comprehend how they arrive at their conclusions.

As a result, the network may perform well on a particular dataset yet struggle when faced with novel data or conditions. This becomes more important when the algorithm’s main function is to produce a rapid and dependable answer to aid clinicians in clinical diagnosis. Since deep neural networks are still largely a mystery, it is important that future research in this area focus on elucidating the factors that may influence them to make a certain decision when presented with several options. Hassani et al. [37] provides a novel alternative to the conventional patching and embedding method by taking advantage of convolutional networks’ capacity to identify salient features from pictures and feed that data into a Transformer. One alternative approach to discovering what causes a network to classify a particular picture in a specific manner is to examine how the data is first partitioned and then to compare that with the classification job completed by the Transformer. We found that our automated COVID-19 identification approach achieved higher accuracy compared to existing methods across all three chest X-ray images.

## Figures and Tables

**Table 1:** CXR images availability from openly available databases.

Dataset	COVID	Pneumonia	Normal
COVID chestxray set [30]	521	239	218
COVID-19 radiography database [31]	3616	1345	10,192
Actualmed COVID-19 chest X-ray dataset [32]	12	0	80
Total CXR images	419	1584	10,490

**Table 2:** Classes and models for classification.

File Name	Class Number	Model Output
COVID-1	1	COVID
COVID-2	1	COVID
COVID-3 (etc.)	1	COVID
Normal-1	2	Normal
Normal-2	2	Normal
Normal-3 (etc.)	2	Normal
Pneumonia-1	3	Pneumonia
Pneumonia-2	3	Pneumonia
Pneumonia-3 (etc.)	3	Pneumonia

**Table 3:** Performance evaluation of CNN architectures.

Network Architecture	Test Accuracy
Inception v3	0.7936
Xception	0.8362
ResNet50	0.8558

**Table 4:** Efficiency parameters for EfficientNetB7 on the training dataset.

Class	Precision (%)	Recall (%)	F1-Score (%)	Support
COVID-19	0.90	0.95	0.92	941
Normal	0.81	0.84	0.82	941
Viral Pneumonia	0.89	0.82	0.85	941

**Table 5:** Efficiency parameters for EfficientNetB7 on the validation dataset.

Class	Precision (%)	Recall (%)	F1-Score (%)	Support
COVID-19	0.85	0.90	0.87	202
Normal	0.81	0.81	0.81	202
Viral Pneumonia	0.83	0.77	0.80	202

**Table 6:** Efficiency parameters for EfficientNetB7 on the testing dataset.

Class	Precision (%)	Recall (%)	F1-Score (%)	Support
COVID-19	0.85	0.93	0.89	202
Normal	0.78	0.81	0.79	202
Viral Pneumonia	0.78	0.68	0.72	202

**Table 7:** Accuracy per class for the training dataset on EfficientNetB7 parameters.

	Training Dataset		Validation Dataset		Testing Dataset	
Class	Acc.	Samples	Acc.	Samples	Acc.	Samples
COVID-19	0.95	142	0.90	31	0.94	31
Normal	0.84	142	0.81	31	0.81	31
Pneumonia	0.82	142	0.77	31	0.68	31

## Data Availability

The data supporting the outcomes of this study are publicly available on Kaggle and have been addressed in Section 3 (i.e., the CXR-DNN technique of the manuscript). Researchers can access the dataset directly through Kaggle to reproduce or build upon our work.
